# Interpretation of vitamin B-12 and folate concentrations in population-based surveys does not require adjustment for inflammation: Biomarkers Reflecting Inflammation and Nutritional Determinants of Anemia (BRINDA) project

**DOI:** 10.1093/ajcn/nqz303

**Published:** 2020-04-08

**Authors:** Melissa F Young, Junjie Guo, Anne Williams, Kyly C Whitfield, Sabiha Nasrin, Vijaya Kancherla, Parminder S Suchdev, Krista S Crider, Christine M Pfeiffer, Mary Serdula

**Affiliations:** 1 Rollins School of Public Health, Emory University, Atlanta, GA, USA; 2 Centers for Disease Control and Prevention, Atlanta, GA, USA; 3 Department of Applied Human Nutrition, Mount Saint Vincent University, Halifax, Canada; 4 ICDDR, B Dhaka, Bangladesh

**Keywords:** inflammation, vitamin B-12, RBC folate, serum folate, BRINDA

## Abstract

**Background:**

Vitamin B-12 and folate deficiencies in women and children have important public health implications. However, the evidence is conflicting and limited on whether the influence of inflammation on biomarker concentrations may be sufficiently and consistently influenced by inflammation to require adjustment for interpreting concentrations or estimating population prevalence of deficiencies.

**Objective:**

We examined correlations between concentrations of the inflammation biomarkers C-reactive protein (CRP) and α1-acid glycoprotein (AGP) and serum vitamin B-12 and serum and RBC folate among nonpregnant women of reproductive age (WRA; 15–49 yr) and preschool children (PSC; 6–59 mo).

**Methods:**

We analyzed cross-sectional data from 16 nationally representative nutrition surveys conducted in WRA (*n* = 32,588) and PSC (*n* = 8,256) from the Biomarkers Reflecting Inflammation and Nutritional Determinants of Anemia project. Spearman correlations between CRP or AGP and vitamin B-12 or folate concentrations were examined, taking into account complex survey design effects.

**Results:**

Correlations between inflammation and vitamin B-12 or folate were weak, with no clear pattern of association in either WRA or PSC. Correlation coefficients between CRP and vitamin B-12 for WRA and PSC ranged from −0.25 to 0.16, and correlations between AGP and vitamin B-12 ranged between −0.07 and 0.14. Similarly, correlations between CRP and serum folate ranged from −0.13 to 0.08, and correlations between AGP and serum folate between −0.21 and 0.02. Only 3 surveys measured RBC folate, and among them, correlations for WRA ranged from −0.07 to 0.08 for CRP and −0.04 for AGP (1 country).

**Conclusions:**

Based on the weak and inconsistent correlations between CRP or AGP and vitamin B-12 or folate biomarkers, there is no rationale to adjust for inflammation when estimating population prevalence of vitamin B-12 or folate deficiencies in WRA or PSC.

## Introduction

Vitamin B-12 and folate are essential micronutrients and their deficiencies have public health implications (1–3). In regions where consumption of animal-source or fortified foods are not common or prohibitively expensive, individuals may be at risk of vitamin B-12 deficiency (4). Malabsorption through pernicious anemia and atrophic gastritis achlorhydria, among other factors, also contributes to vitamin B-12 deficiency (5). Adequate folate status is of particular importance among women of reproductive age (WRA) because low-folate status in early pregnancy increases the risk of neural tube defects among offspring (6–8). However, despite the importance of these nutrients, their measurement is generally not included in population surveys, and data gaps preclude global estimates of vitamin B-12 and folate deficiencies (1, 3, 9). For folate in particular, data availability is complicated by the fact that there are large differences in folate assays due to different assay approaches (e.g., microbiological, immunoassay, and chromatography-based), analytes (e.g., total compared with major circulating types of folate), and antibodies used for immunoassay approaches, making it difficult to choose the correct thresholds to define deficiency in many situations (10–12).

In addition, there is limited evidence on the question of whether folate or vitamin B-12 may be influenced by inflammation and if this should be taken into account when estimating the prevalence of deficiency (13–15). Inflammation is common in many settings where nutritional assessment occurs and is assessed frequently in large surveys by measuring acute-phase proteins C-reactive protein (CRP) and α1-acid glycoprotein (AGP) (16). When assessing the nutritional status of a population using biomarker surveys it is critical to understand the interaction of inflammation with the biomarkers of interest. Recent findings from the Biomarkers Reflecting Inflammation and Nutritional Determinants of Anemia (BRINDA) project demonstrated relations between biomarkers of inflammation and biomarkers of both iron and vitamin A and recommended methods for adjustment in settings of inflammation (17–21). However, there has been limited systematic exploration of the potential role of inflammation on biomarkers of vitamin B-12 and folate in large-scale surveys. For adjustments to be recommended, there should be evidence of biological plausibility that inflammation is acting independently of dietary intake and consistency in relations between micronutrient biomarkers and inflammation across a variety of contexts. A review by Tomkins describes a reduction in serum folate during acute inflammation, while RBC folate appears to be more stable (reflecting mean folate status during proceeding ∼120 days) and less affected by acute inflammatory responses (15). McMillan et al. report an inverse relation with vitamin B-12 and CRP in clinical settings (22). However, there remains conflicting evidence surrounding the impact of inflammation on biomarkers of these nutrients (13–15, 23, 24). To address these gaps in our understanding our objective was to examine if inflammation is correlated with biomarkers of vitamin B-12 and folate status among WRA and preschool children (PSC) in the BRINDA project.

## Methods

We used cross-sectional survey data from the BRINDA project (www.BRINDA-nutrition.org). A description of the methods used to identify data sets, define inclusion and exclusion criteria, and manage the BRINDA database has been previously reported (19, 21). In brief, we included nationally representative surveys in PSC (aged 6–59 mo), nonpregnant WRA (15–49 yr), or both where data were available for ≥1 biomarker of inflammation (CRP, AGP, or both) and ≥1 biomarker for vitamin B-12 or folate (serum or RBC folate) (**Supplemental Figure 1**).

### Laboratory analysis

Venous blood was obtained from all survey participants, with the exception of participants from Mongolia, from whom there was a combination of capillary and venous blood samples. Plasma and serum samples were stored at −20°C until analysis in all participating surveys. Laboratory methods used for analysis of CRP, AGP, vitamin B-12, serum folate, and RBC folate are summarized in **Supplemental Table 1** for each survey. Given large discrepancies between commercially available folate assays (11), we did not pool folate data across surveys for meta-analyses. Differences in vitamin B-12 results among assays also exist (25); thus, we chose not to pool vitamin B-12 results across surveys either. Additionally, we did not report on estimated prevalence of deficiencies, given the wide variation in laboratory methods. Inflammation was defined according to a CRP concentration >5 mg/L or an AGP concentration >1 g/L (26). Methods for CRP and AGP assessment are mostly consistent across assays due to availability of reference materials and external quality assessment programs (27–31). Malaria was measured in 3 WRA and 1 PSC surveys. Malaria infection was defined as either positive or negative based on rapid diagnostic kit results.

### Statistical analysis

All statistical analyses were performed using SAS 9.4 (SAS Institute). All analyses took into account complex survey design effects, including cluster, strata, and biomarker-specific sampling weights, unless otherwise specified. The weighted Spearman's correlation coefficients were determined by first calculating the ranks of inflammation (CRP or AGP) and vitamin B-12 or folate (serum or RBC) within each survey and then estimating the weighted Pearson's correlation between the rank variables. Unweighted correlation analyses were also conducted and produced similar findings (data not shown). The geometric means and 95% CIs of vitamin B-12 and folate measure were plotted by CRP and AGP decile within each survey to investigate if there was a linear relation between inflammation and vitamin B-12 or folate, similar to prior BRINDA publications (19). Results were considered significant at *P* < 0.05. Analyses were conducted separately for each survey.

Sensitivity analyses were conducted with surveys to examine the potential role of malaria infection, because folate is required for parasite replication. To investigate the interaction of malaria infection with nutrient biomarkers, we stratified individuals by malaria status in the 3 surveys that contained individual-level malaria information, compared stratified correlations, and tested for interactions. Although we also intended to explore the role of fasting, insufficient individual-level data on fasting data to test the interaction of fasting were available; only 1 survey had both fasted and nonfasted subjects, and in this survey few subjects were fasted (*n* = 13) (data not shown).

## Results

### Participant characteristics

Our study sample was restricted to participants with no missing values for either vitamin B-12, serum folate, or RBC folate, and with data available for CRP or AGP, or both. On the basis of these criteria, data on WRA were available from 14 surveys, and on PSC in 8 surveys, representing 32,588 WRA and 8256 PSC. Vitamin B-12 data were available from 13 surveys in WRA (*n* = 29,552) and 5 surveys in PSC (*n* = 5726). For serum folate, 12 surveys from WRA (*n* = 30,900) and 8 surveys from PSC (*n* = 8132) were available. RBC folate was measured in only 3 surveys in WRA (*n* = 12,048) and 2 surveys in PSC (*n* = 3318). Mean age among PSC ranged from 20.1 mo in Mongolia to 41.8 mo in Mexico (**Table 1**). Among WRA, mean age ranged from 27.1 yr in Cameroon to 34.7 yr in Mexico. The prevalence of elevated CRP ranged from 6.0% to 38.4% in PSC and from 5.3% to 27.9% in WRA. The prevalence of elevated AGP ranged from 24.6% to 39.8% in PSC and from 5.5% to 33.6% in WRA. **Table 2** reports median and IQRs for vitamin B-12, serum folate, and RBC folate.

**TABLE 1:** Age and inflammation of PSC and nonpregnant WRA, BRINDA project^1^

			CRP		AGP	
Survey, year	*n*	Age,^2^ mean (95% CI)	Mean (95% CI), mg/L	CRP concentration >5 mg/L, mean (95% CI), %	Mean (95% CI), mg/L	AGP concentration >1 g/L, mean (95% CI), %
PSC						
Cambodia, 2014	646	36.4 (35.3, 37.5)	2.2 (1.5, 3)	10.1 (7.3, 12.9)	1.1 (1, 1.3)	36 (29.4, 42.6)
Cameroon, 2009	362	32.3 (30.9, 33.8)	5.6 (4.7, 6.4)	38.4 (31.3, 45.4)	1 (0.9, 1)	39.8 (32.1, 47.4)
Ecuador, 2012	2019	30.8 (29.5, 32.2)	4 (3.5, 4.5)	12.5 (10.1, 14.9)	—	—
Mexico, 2006	974	41.8 (40.6, 42.9)	2.2 (1.7, 2.6)	10.3 (7.6, 13)	—	—
Mexico, 2012	2538	39.1 (38.5, 39.7)	2.4 (2, 2.9)	11.7 (9.3, 14.1)		
Mongolia, 2006	228	20.1 (20.1, 21.3)			0.8 (0.8, 0.9)	24.6 (19.0, 30.2)
USA, 2006	1315	36.7 (35.6, 37.8)	1.4 (1.1, 1.7)	6 (4.5, 7.5)	—	—
Vietnam, 2010	174	38.5 (36.9, 40.1)	2.4 (1.4, 3.5)	12.6 (7.8, 17.5)	—	—
WRA						
Azerbaijan, 2013	2611	32.1 (31.6, 32.5)	2.8 (2.5, 3)	13.2 (11.2, 15.1)	0.9 (0.9, 0.9)	31.7 (29.3, 34)
Bangladesh, 2012	863	30 (28.8, 31.1)	1.5 (1.2, 1.8)	5.3 (2.8, 7.8)	0.7 (0.7, 0.8)	13.2 (9.1, 17.3)
Cambodia, 2014	700	30.2 (29.4, 30.9)	2 (1.6, 2.3)	9.5 (7.2, 11.9)	1 (0.9, 1.1)	33.6 (24.9, 42.3)
Cameroon, 2009	335	27.1 (26.2, 28)	2.7 (2.2, 3.1)	15.8 (11.4, 20.3)	0.8 (0.7, 0.8)	5.5 (2.6, 8.4)
Colombia, 2010	611	29.1 (28.1, 30.2)	5.4 (4.3, 6.5)	26.5 (21.9, 31.2)	—	—
Côte d'Ivoire, 2007	792	27.6 (26.9, 28.3)	3.8 (3.2, 4.3)	19.7 (16.5, 22.9)	0.9 (0.8, 0.9)	26.5 (22.9, 30)
Ecuador, 2012	8118	30.3 (29.8, 30.7)	4.1 (3.9, 4.3)	17.4 (16, 18.8)	—	—
Georgia, 2009	407	34.2 (33.2, 35.3)	5.2 (4.3, 6.2)	27.9 (23.3, 32.5)	—	—
Malawi, 2016	767	28.4 (27.7, 29)	2.4 (1.7, 3.1)	7.6 (5.3, 9.9)	0.7 (0.6, 0.7)	11.1 (8, 14.3)
Mexico, 2012	3608	34.7 (34.4, 35.1)	3.7 (3.4, 4)	20.8 (18.2, 23.3)	—	—
Pakistan, 2011	8307	30.8 (30.6, 30.9)	2.7 (2.4, 2.9)	11.6 (10.7, 12.5)	0.8 (0.8, 0.9)	23.9 (22.5, 25.3)
UK, 2014	895	34.4 (33.5, 35.3)	3.3 (3, 3.7)	16 (12.7, 19.2)	—	—
USA, 2006	3197	33.5 (33, 34)	4.4 (4, 4.8)	25.6 (23.5, 27.8)	—	—
Vietnam, 2010	1377	32.2 (31.6, 32.7)	1.8 (1.6, 2.1)	6.5 (5.3, 7.7)	—	—

1 AGP, α1-acid glycoprotein; BRINDA, Biomarkers Reflecting Inflammation and Nutritional Determinants of Anemia; CRP, C-reactive protein; PSC, preschool children; WRA, women of reproductive age.

2 Age values are shown in months for preschool children and years for women of reproductive age. Mongolia did not apply complex survey design.

**TABLE 2:** Vitamin B-12, serum folate, and RBC folate in PSC and nonpregnant WRA: BRINDA project^1^

Survey, year	Vitamin B-12, *n*	Vitamin B-12, median (25th, 75th percentile), pmol/L	Serum folate sample size, *n*	Serum folate, median (25th, 75th percentile), pmol/L	RBC folate, *n*	RBC folate median (25th, 75th percentile), nmol/L
PSC						
Cambodia, 2014	645	413.3 (312.3, 597.8)	646	20.9 (14.8, 29.2)	—	—
Cameroon, 2009	361	312.1 (195.6, 493.2)	362	21.2 (14.4, 30.1)	—	—
Ecuador, 2012	—	—	2019	57.4 (42.6, 77.3)	2019	1029.2 (786.4, 1325.6)
Mexico, 2006	931	461.1 (315.6, 642.7)	940	28.7 (20.4, 37.7)	—	—
Mexico, 2012	2527	526.4 (381.5, 693.4)	2496	35.9 (32.0, 38.8)	—	—
Mongolia, 2006	—	—	228	17.7 (12.3, 24.6)		—
USA, 2006	1262	606.7 (462.8, 796.6)	1267	35.8 (25.0, 44.7)	1299	597.6 (498.9, 729.2)
Vietnam, 2010	—	—	174	22.0 (17.3, 29.1)		—
WRA						
Azerbaijan, 2013	1317	272.2 (182.7, 345.7)	2551	10.9 (9.4, 13.0)	—	—
Bangladesh, 2012	863	311.8 (229.7, 413.3)	847	10.6 (8.6, 13.9)	—	—
Cambodia, 2014	700	498.3 (359.6, 690.9)	699	13.8 (10.6, 18.7)	—	—
Cameroon, 2009	335	343.7 (208.8, 547.6)	335	17.8 (11.5, 26.4)	—	—
Colombia, 2010	611	275.7 (196.8, 473.9)	—	—	—	—
Côte d'Ivoire, 2007	400	300.9 (187.9, 447.6)	792	3.6 (2.1, 6.5)	—	—
Ecuador, 2012	8116	401.5 (302.7, 553.3)	8118	32.3 (22.9, 45.3)	8118	895.5 (699.4, 1182.7)
Georgia, 2009	—	—	407	5.2 (3.1, 8.4)	—	—
Malawi, 2012	765	252.7 (180.8, 375.6)	755	18.0 (11.3, 29.1)	752	492.8 (352.0, 668.1)
Mexico, 2012	3608	366.1 (279.6, 530.9)	3608	29.3 (22.4, 33.0)	—	—
Pakistan, 2011	8287	141.1 (62.8, 291.2)	8257	8.7 (2.8, 40.3)	—	—
UK, 2014	895	243.6 (197.7, 306.3)	—	—	—	—
USA, 2006	3166	328.0 (252.7, 436.5)	3186	24.9 (18.3, 32.8)	3178	562.5 (448.5, 707.6)
Vietnam, 2010	489	552.5 (329.0, 881.0)	1345	16.8 (12.5, 22.7)	—	—

1 Mongolia did not apply a complex survey design. BRINDA, Biomarkers Reflecting Inflammation and Nutritional Determinants of Anemia; PSC, preschool children; WRA, women of reproductive age.

### Relation between vitamin B-12 or folate biomarkers and inflammation

Strong positive correlations between CRP and AGP were found across data sets that had both inflammation biomarkers (correlation coefficient ranged from 0.2 to 0.7, *P* < 0.0001, data not shown). Weak and inconsistent correlation coefficients between vitamin B-12 and inflammation biomarkers (CRP and AGP) were observed for WRA and PSC (**Table 3**). For WRA, 3 surveys reported significant positive associations between vitamin B-12 and CRP, 3 surveys reported significant negative associations, and in 7 surveys, the correlations were nonsignificant. Correlations between vitamin B-12 and AGP were similar, with 3 surveys reporting significant positive associations and 4 surveys reporting nonsignificant associations. Likewise, among PSC there were no clear patterns of associations for vitamin B-12 and CRP or AGP, with correlation coefficients ranging from 0.12 to −0.07.

**TABLE 3:** Weighted Spearman correlations between vitamin B-12 and CRP and AGP in PSC and nonpregnant WRA, BRINDA project^1^

		Vitamin B-12 and CRP correlation			Vitamin B-12 and AGP correlation	
Survey, year	*n*	*r*	*P*	*n*	*r*	*P*
PSC						
Cambodia, 2014	645	−0.01	0.752	645	−0.07	0.283
Cameroon, 2009	361	0.00	0.967	361	−0.06	0.311
Mexico, 2006	931	0.00	0.946		—	—
Mexico, 2012	2527	0.05	0.146		—	—
USA, 2006	1262	0.12	0.001		—	—
WRA						
Azerbaijan, 2013	1317	−0.02	0.629	1317	−0.02	0.586
Bangladesh, 2012	862	0.02	0.408	863	0.14	0.001
Cambodia, 2014	700	−0.01	0.843	700	−0.05	0.350
Cameroon, 2009	335	0.01	0.802	335	−0.03	0.566
Colombia, 2010	611	−0.25	0.307		—	—
Côte d'Ivoire, 2007	400	0.16	0.008	400	0.14	0.005
Ecuador, 2012	8116	0.00	0.791		—	—
Malawi, 2016	765	0.01	0.744	765	0.00	0.958
Mexico, 2012	3608	0.10	0.001		—	—
Pakistan, 2011	8287	0.04	0.017	6509	0.04	0.034
UK, 2014	895	−0.11	0.020		—	—
USA, 2006	3166	−0.08	<0.001		—	—
Vietnam, 2010	489	−0.09	0.032		—	—

1 *P* value was calculated from a *t* test in a regression model that took into account complex sampling (cluster, strata, and sampling weight). AGP, α1-acid glycoprotein; BRINDA, Biomarkers Reflecting Inflammation and Nutritional Determinants of Anemia; CRP, C-reactive protein; PSC, preschool children; WRA, women of reproductive age.

**Table 4** presents the summary of the correlations between serum folate and CRP and AGP among WRA and PSC. For WRA, the correlations between serum folate and CRP ranged from −0.13 to 0.08, with a significant positive correlation in 1 survey, significant negative correlations in 4 surveys, and nonsignificant results in 7 surveys. For AGP, correlations with serum folate in WRA ranged from −0.12 to 0.02, with significant negative correlations in 4 surveys and nonsignificant results in 3 surveys. Results were similar among PSC, with the majority of surveys reporting nonsignificant correlations between serum folate and CRP and AGP. Only 1 survey showed a significant positive correlation with serum folate and CRP, and 1 showed a significant negative correlation with serum folate and AGP.

**TABLE 4:** Weighted Spearman correlations between serum folate and CRP and AGP in PSC and nonpregnant WRA, BRINDA project^1^

		Serum folate and CRP correlation			Serum folate and AGP correlation	
Survey, year	*n*	*r*	*P*	*n*	*r*	*P*
PSC						
Cambodia, 2014	646	−0.05	0.319	646	−0.21	<0.001
Cameroon, 2009	362	0.00	0.948	362	−0.04	0.555
Ecuador, 2012	2019	0.05	0.005		—	—
Mexico, 2006	940	0.01	0.845		—	—
Mexico, 2012	2496	−0.04	0.246		—	—
Mongolia, 2006		—	—	228	−0.07	0.292
USA, 2006	1267	−0.06	0.100		—	—
Vietnam, 2010	174	−0.06	0.506	—	—	—
WRA						
Azerbaijan, 2013	2551	0.07	0.008	2551	0.00	0.992
Bangladesh, 2012	846	−0.09	0.091	847	−0.12	0.028
Cambodia, 2014	699	−0.07	0.198	699	−0.12	0.032
Cameroon, 2009	335	−0.06	0.329	335	0.02	0.641
Côte d'Ivoire, 2007	792	−0.09	0.003	792	0.00	0.909
Ecuador, 2012	8118	−0.12	<0.001		—	—
Georgia, 2009	407	0.08	0.088		—	—
Malawi, 2016	755	−0.13	0.013	755	−0.11	0.026
Mexico, 2012	3608	0.03	0.242		—	—
Pakistan, 2011	7819	0.02	0.135	6481	−0.04	0.017
USA, 2006	3186	−0.07	0.003		—	—
Vietnam, 2010	1345	0.02	0.402		—	—

1 *P* value was calculated from a *t* test in a regression model that took into account complex sampling (cluster, strata, and sampling weight). Mongolia did not apply a complex survey design. AGP, α1-acid glycoprotein; BRINDA, Biomarkers Reflecting Inflammation and Nutritional Determinants of Anemia; CRP, C-reactive protein; PSC, preschool children; WRA, women of reproductive age.

Survey data that contained both RBC folate and markers of inflammation were limited (**Table 5**). Among the 3 surveys that assessed RBC among WRA, 2 resulted in weak significant positive correlations with CRP, and in 1 survey the relation was nonsignificant. Only 1 survey had data available on RBC folate and AGP, and in this survey the correlations were nonsignificant. Among PSC, data were available for 2 surveys and there was a weak significant negative correlation (−0.07) in survey results from 1 country and nonsignificant results in the second survey. Data were not available to assess correlations between RBC folate and AGP in PSC.

**TABLE 5:** Weighted Spearman correlations between RBC folate and CRP and AGP in PSC and non-pregnant WRA, BRINDA project^1^

		RBC folate and CRP correlation			RBC folate and AGP correlation	
Survey, year	*n*	*r*	*P*	*n*	*r*	*P*
PSC						
Ecuador, 2012	2019	−0.07	0.045		—	—
USA, 2006	1299	−0.01	0.833		—	—
WRA						
Ecuador, 2012	8118	0.05	0.006		—	—
Malawi, 2016	752	−0.06	0.283	752	−0.04	0.432
USA, 2006	3178	0.08	0.003		—	—

1 *P* values were calculated from a *t* test in a regression model that took into account complex sampling (cluster, strata, and sampling weight). AGP, α1-acid glycoprotein; BRINDA, Biomarkers Reflecting Inflammation and Nutritional Determinants of Anemia; CRP, C-reactive protein; PSC, preschool children; WRA, women of reproductive age.

The relations between the mean nutrient concentrations for vitamin B-12, serum folate, and RBC folate and inflammation deciles (CRP and AGP) were highly variable, with no clear pattern of association across the surveys (**Supplemental Figures 2–12**). To examine the potential role of malaria status on the aforementioned correlations, we conducted a stratified analysis and tested for interaction among the 3 surveys with individual data on malaria and inflammation and vitamin B-12, serum folate, or RBC folate (**Supplemental Table 2**). For vitamin B-12, associations with CRP remained weak regardless of malaria status and there was no evidence of interaction in WRA or PSC. For vitamin B-12 and AGP, there was a significant interaction among WRA in Malawi; women who were infected by malaria had a negative correlation (−0.21), whereas those without malaria had a positive association (0.05). However, results were nonsignificant in Côte d'Ivoire and Cameroon. For serum folate and RBC folate there was no evidence of interaction with CRP or AGP in either WRA or PSC.

## Discussion

In our large, multicountry analysis of approximately 32,000 WRA and 8000 PSC, there was no clear pattern of association between inflammation (CRP or AGP) and vitamin B-12 and serum or RBC folate among WRA and PSC. Correlations were weak (most <0.2) and of various levels of significance and direction (some positive and some negative). In addition, there was no clear pattern of association between inflammation deciles (CRP and AGP) and micronutrient biomarkers across the surveys. Thus, current data do not support the need to adjust these biomarkers for inflammation in population-based surveys. However, there remain important data gaps, in particular for RBC folate, AGP, and child-level data, that may merit re-examination when further data are available.

The weak and inconsistent correlations among the 16 surveys included in the BRINDA data sets are in alignment with the conflicting existing research on this topic (13–15, 23, 24, 32, 33). While deciding when and if to adjust a nutrient biomarker for inflammation, it is important to first review if there is clearly a biological rationale to justify adjustment. For example, there is a strong biological rationale for adjusting serum ferritin, which itself is an acute-phase response protein, for inflammation (18). There is evidence, although inconsistent, that inflammation could affect folate status through enteric malabsorption, increasing folate requirements to support erythropoiesis in the presence of repeated malarial infections, among other mechanisms that remain not fully understood (24, 34). Conversely, folate insufficiency may affect inflammation through multiple mechanisms (24, 35–37). For example, low folate may impair methionine synthase activity, leading to hyperhomocysteinemia and damage to endothelial cells (24, 35–37). Likewise, there is some evidence of a bidirectional relation with vitamin B-12 and inflammation or infection through the following potential mechanisms: chronic inflammatory conditions such as hepatitis may increase serum transcobalamins and vitamin B-12, altered serum concentrations may result from cytolysis of liver cells and impaired clearance as a result of liver diseases, and infections may impair absorption through reduced secretion of gastric acid or competition for vitamin B-12, among other potential mechanisms in which there is limited human evidence (24, 38, 39). It is important to note that many of these potential mechanisms may explain intermediate pathways between inflammation and folate or vitamin B-12 status. However, there is not a consensus on how inflammation may impact folate or vitamin B-12 concentrations. Thus, even if statistically significant associations exist between micronutrient and inflammation indicators within a given survey, at this time there does not appear to be a clear biological rationale to justify adjustment.

This is one of the largest studies to date to examine correlations between inflammation biomarkers and serum vitamin B-12, serum folate, and RBC folate; however, there remain critical gaps in our understanding due to varying laboratory methods and data availability that merit further examination and future research. The relations between nutrition biomarkers and inflammation may vary based on the severity of deficiency and the underlying prevalence of inflammation. However, in our analysis there were no clear patterns of association. For example, despite differences in elevated CRP in Cameroon (38%) and Mexico, 2006 (10%), there were very similar correlations between vitamin B-12 and CRP (*r* = 0.00, *P* = 0.97; *r* = 0.00, *P* = 9.5) and serum folate (*r* = 0.00, *P* = 0.95; *r* = 0.01, *P* = 0.85), respectively. We were not able to pool the data or compare vitamin blood concentrations across surveys due to method differences across surveys (11, 25, 40). Large differences in results have been reported among assays, 1.5- to 6-fold differences for serum folate and 8- to 40-fold differences in RBC folate (11). Although differences in vitamin B-12 results among assays are smaller (up to ∼2-fold) assay errors and, particularly, an overestimation of low vitamin B-12 concentrations by some assays have been reported (25), we therefore chose not to pool vitamin B-12 results across surveys either. Pooling of biomarker data from these surveys would not be appropriate and would further complicate interpretation of correlations. Laboratory assays for numerous nutritional biomarkers, including folate and vitamin B-12, require standardization and the use of certified reference materials to verify method accuracy and to ensure better comparability of data across laboratories and over time (36, 41). As this is a secondary analysis of existing data, we are limited by the micronutrient and inflammation biomarkers data available in the data sets. Our analysis was particularly limited by a lack of information about CRP in the data sets, for which limits of detection (LODs) and limits of quantification (LOQs) vary widely across assays, and for which there may be high proportions of undetectable/unquantifiable observations. In a sensitivity analysis, surveys conducted at a single laboratory with a known CRP LOD of 0.5 mg/L were assigned a CRP value of 0.25 mg/L, and our correlation analysis was rerun. As shown in **Supplemental Tables 3–5**, the results of this sensitivity analysis yielded similar results to the primary analysis. Unfortunately, information on the LODs and LOQs was not available for all surveys, and future work should investigate how different approaches for handling values below the LOD/LOQ may influence interpretation of biomarkers. Data on dietary intake, availability of fortified foods, and morbidity symptoms, which could explain reasons for deficiency and causes of inflammation, would also be useful for exploring contextual features that were included in the surveys. Few surveys had individual-level malaria or fasting data available, which precludes us from being able to determine the potential influence of these factors on our results. We do not have data on other indicators of folate and vitamin B-12 status (e.g., total homocysteine or methylmalonic acid) or other inflammation biomarkers (e.g., TNF-α or albumin). For example, previous research in Saudi Arabia demonstrated a significant inverse association between vitamin B-12 and TNF-α among adults but not children (42).

Our findings are limited to those from population-based surveys and should not be generalized to the clinical setting where individual patient data are interpreted. Prior clinical-based studies have examined the changes in nutrient levels following surgery and found mixed effects (14, 43). The current findings are also limited to WRA and PSC, and future work is needed to confirm these findings in pregnant women, men, and infants. Lastly, the cross-sectional nature of the surveys included in the present study preclude us from determining causality. The potential for reverse causality cannot be ruled out, given that micronutrients may have proinflammatory or anti-inflammatory effects.

The key strength of this study is the large and diverse data set for analysis. We had data on 32,588 WRA and 8256 PSC from across the globe. This diversity was critical for providing a more comprehensive picture and also suggests caution should be taken before making recommendations from one data set alone. With our large sample sizes, we are more likely to detect statistically significant results. For example, while the relation between CRP and vitamin B-12 was statistically significant in Ecuador (*n* = 8118), the correlation was only 0.03 and unlikely to be of biological significance. Given the limitations of this work, we were conservative in our analysis and interpretation of the findings. This study highlights the need to establish a clear biological rationale and consistent correlations across countries and populations before applying the BRINDA approach to adjust nutrient biomarkers for inflammation.

In conclusion, our current analysis of 16 nationally representative surveys does not support the need to adjust vitamin B-12, serum folate, or RBC folate for inflammation in population-based surveys of women of reproductive age or preschool children. There is a need to reexamine this question as progress is made in standardizing laboratory methods across regions and as data with new or more sensitive biomarkers become available.

## Supplementary Material

nqz303_Supplemental_File

## Notes

Supported by the Bill & Melinda Gates Foundation grant OPP1158306, Centers for Disease Control and Prevention, *Eunice Kennedy Shriver* National Institute of Child Health and Human Development, HarvestPlus, and the United States Agency for International Development.

Supplemental Tables 1–5 and Supplemental Figures 1–12 are available from the “Supplementary data” link in the online posting of the article and from the same link in the online table of contents at https://academic.oup.com/ajcn/.

Abbreviations used: AGP, α1-acid glycoprotein; BRINDA, Biomarkers Reflecting Inflammation and Nutritional Determinants of Anemia; CRP, C-reactive protein; LOD, limit of detection; LOQ, limit of quantitation; PSC, preschool children; WRA, women of reproductive age.
