# Supplementary material for: Interpretation of vitamin B-12 and folate concentrations in population-based surveys does not require adjustment for inflammation: Biomarkers Reflecting Inflammation and Nutritional Determinants of Anemia (BRINDA) project
Source: Am J Clin Nutr. 2020 Apr 8;111(4):919–26. doi: 10.1093/ajcn/nqz303 (PMC7138687; doi:10.1093/ajcn/nqz303)
Supplement: nqz303_Supplemental_File [file nqz303_supplemental_file.pdf]

Manuscript: Young et al., Interpretation of vitamin B-12 and folate status concentrations in population based surveys does not require adjustment for inflammation: Biomarkers Reflecting Inflammation and Nutritional Determinants of Anemia (BRINDA) project

ONLINE SUPPORTING MATERIAL

Supplemental Figure 1: BRINDA Project Available Data for Vitamin B12 and Folate

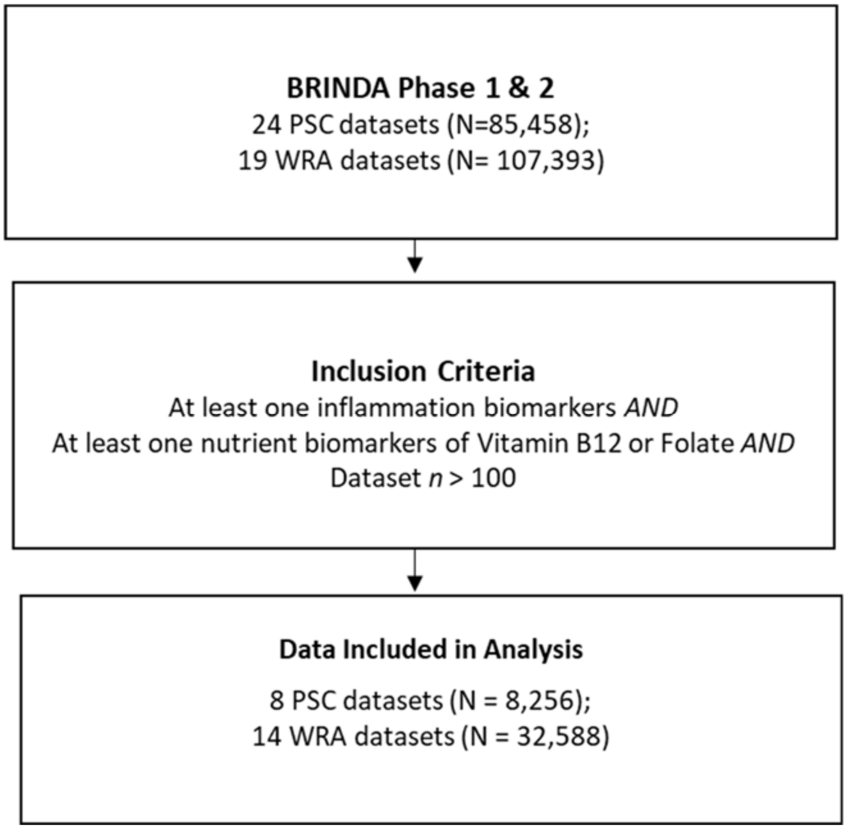

ONLINE SUPPORTING MATERIAL

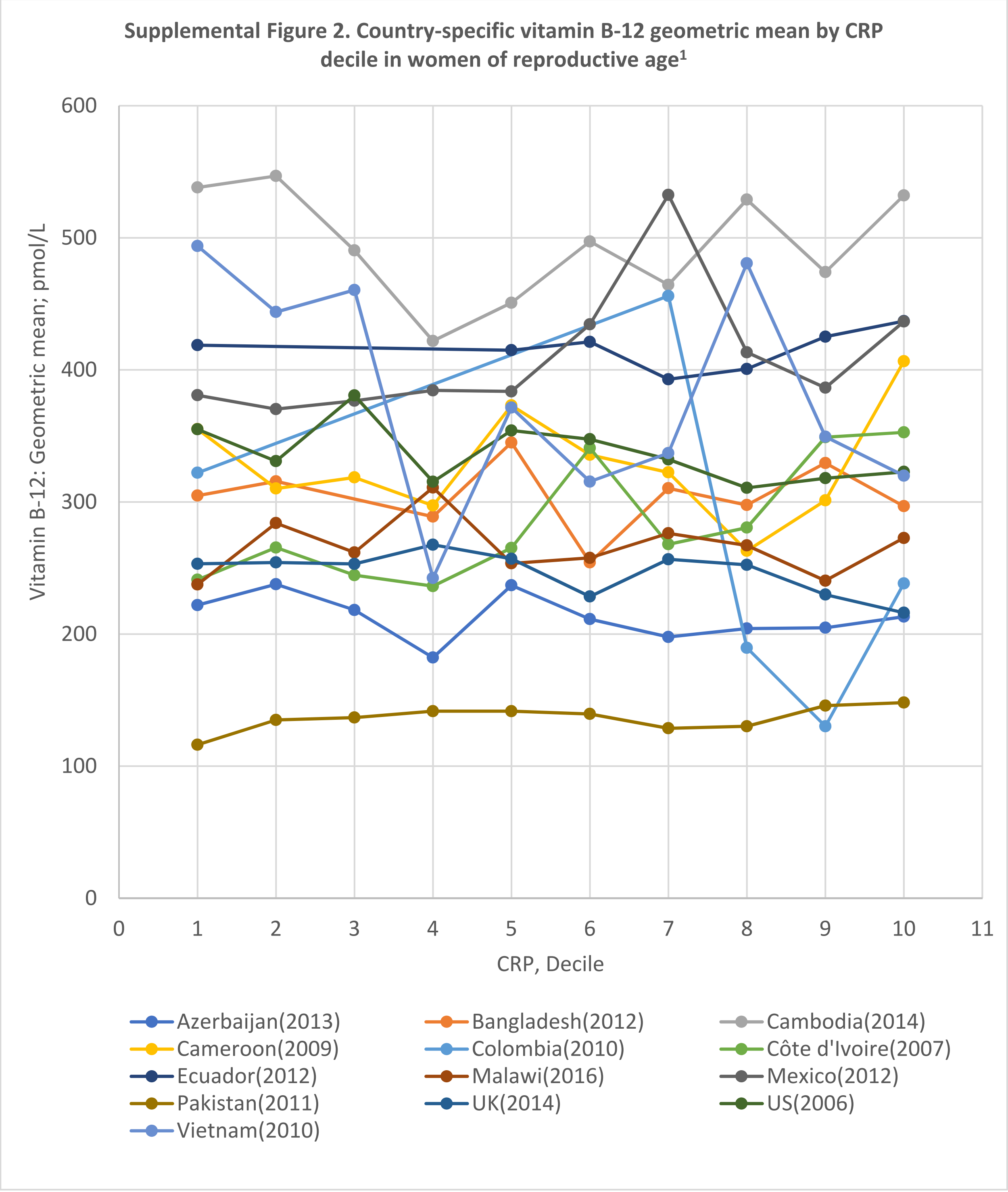

[1]alpha-1-acid glycoprotein: AGP; C-reactive protein: CRP

ONLINE SUPPORTING MATERIAL

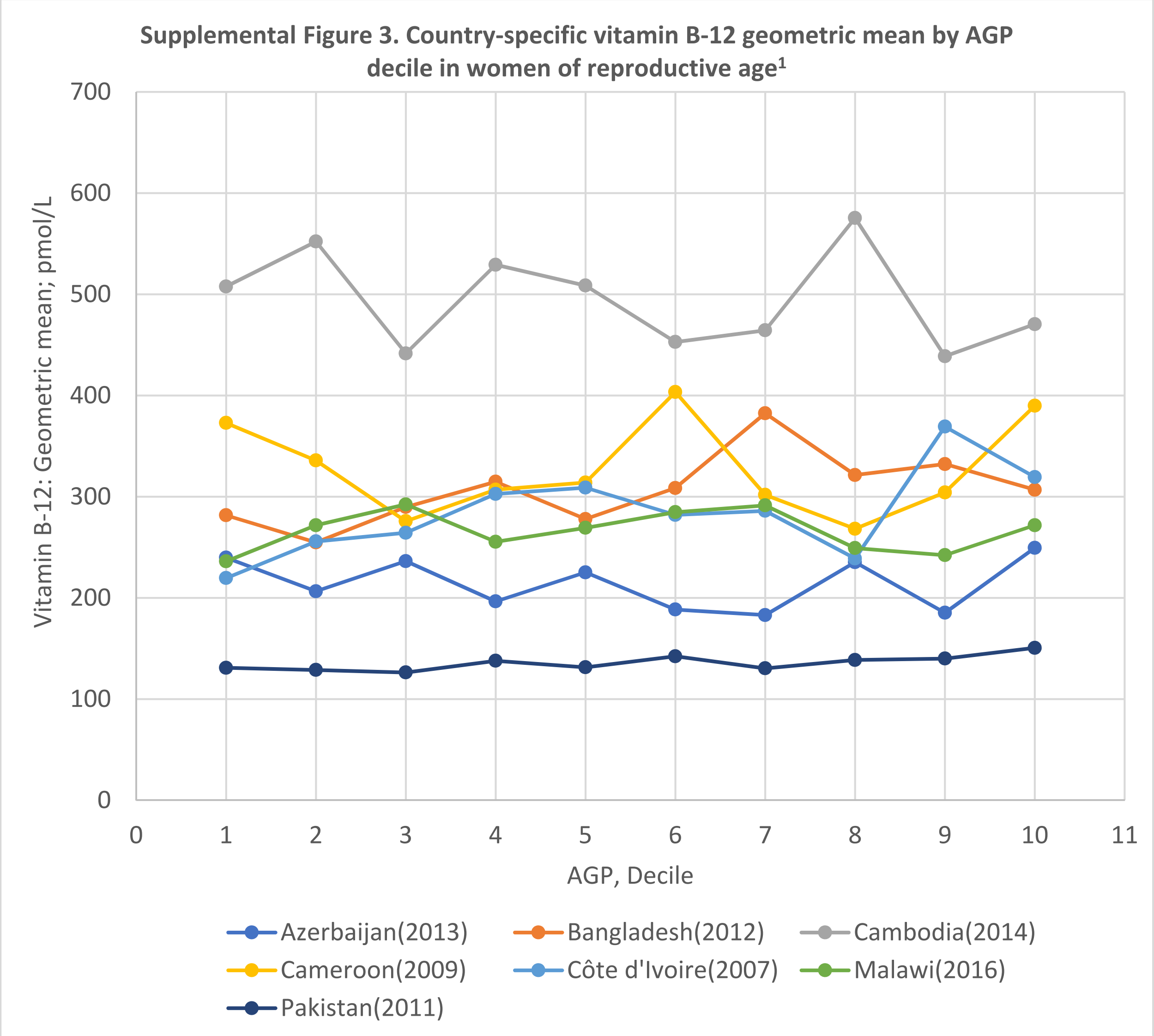

[1]alpha-1-acid glycoprotein: AGP; C-reactive protein: CRP

ONLINE SUPPORTING MATERIAL

Supplemental Figure 4. Country-specific vitamin B-12 geometric mean by CRP decile in preschool children[1]

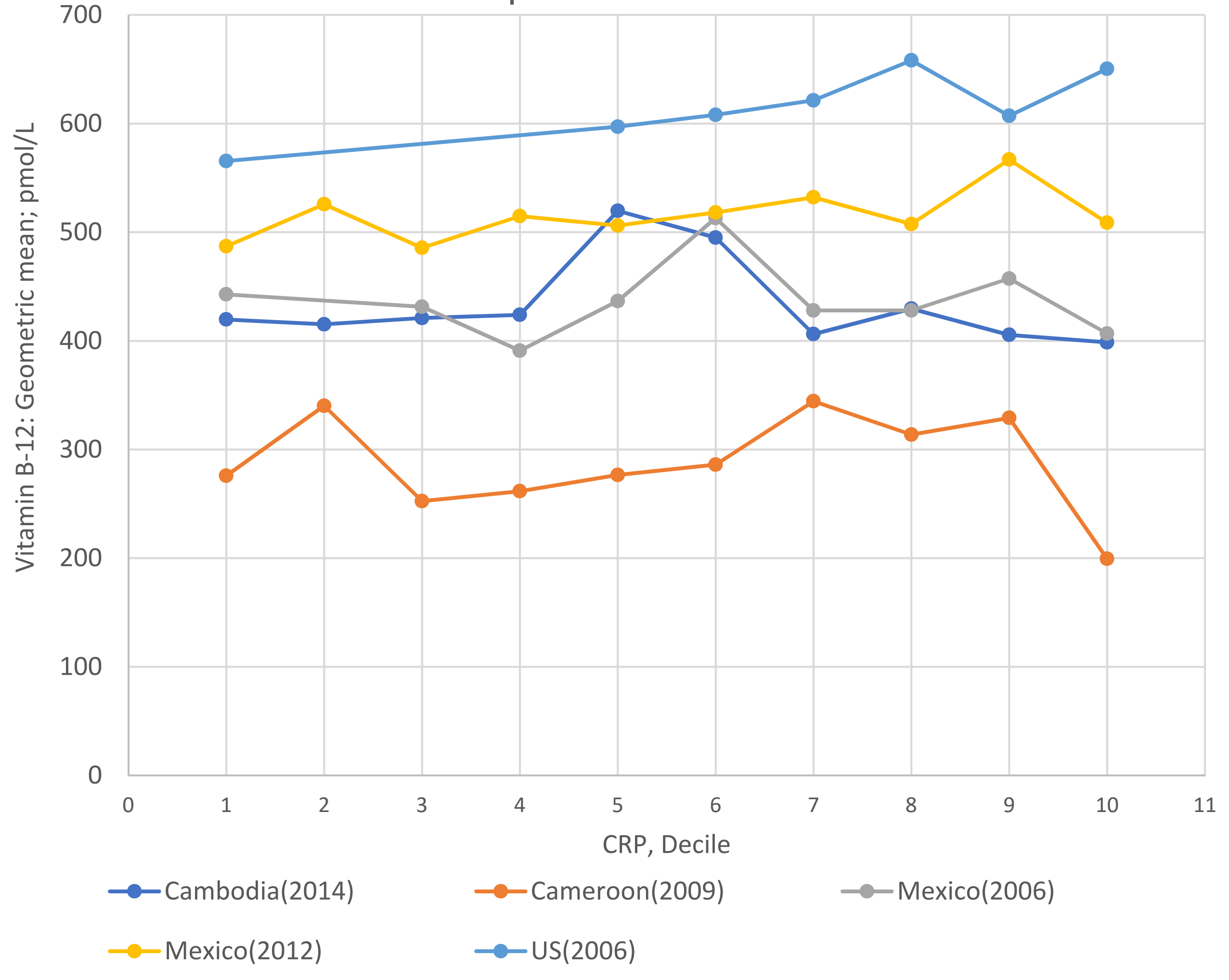

[1]alpha-1-acid glycoprotein: AGP; C-reactive protein: CRP

ONLINE SUPPORTING MATERIAL

Supplemental Figure 5. Country-specific vitamin B-12 geometric mean by AGP decile in preschool children[1]

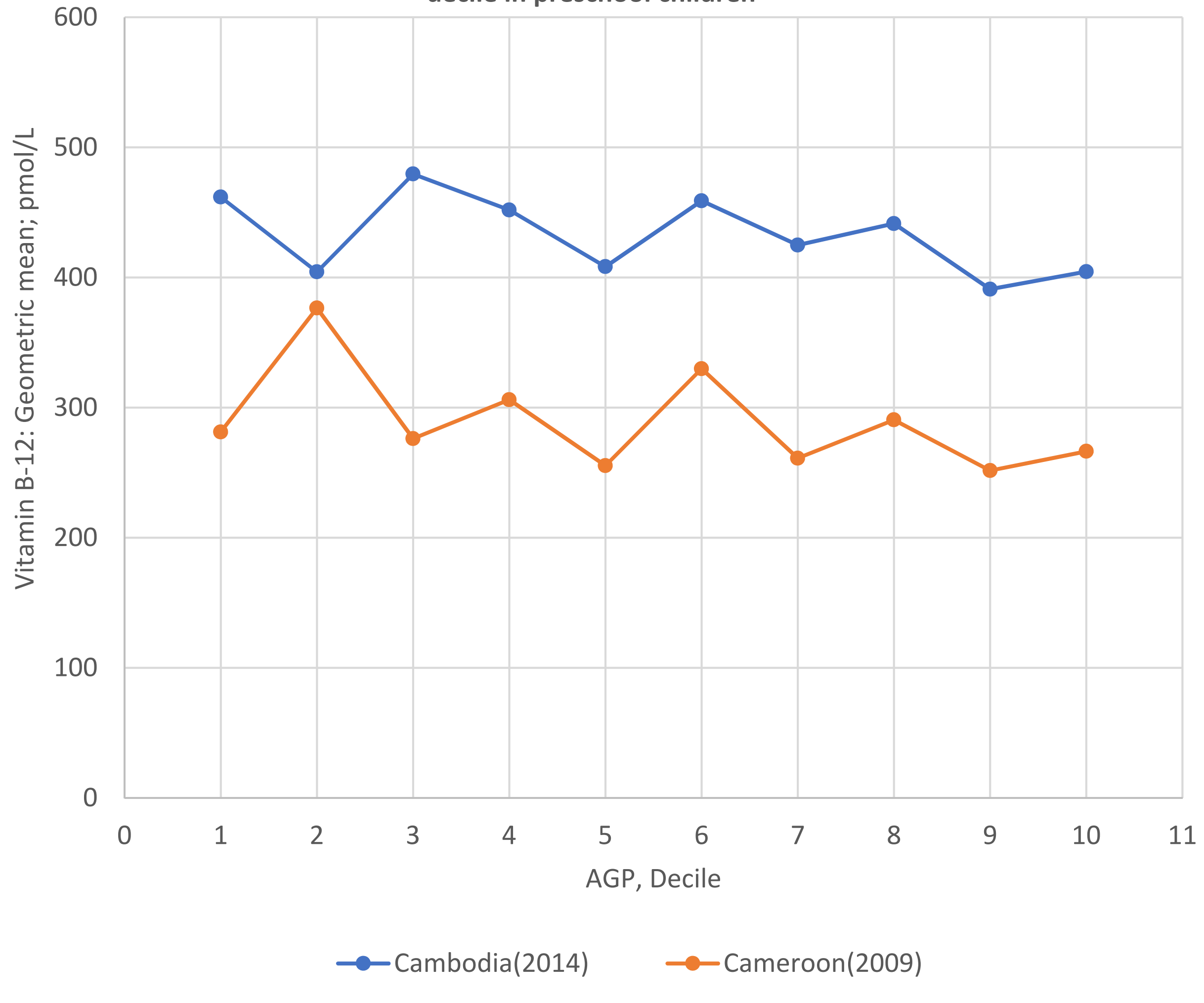

[1]alpha-1-acid glycoprotein: AGP; C-reactive protein: CRP

ONLINE SUPPORTING MATERIAL

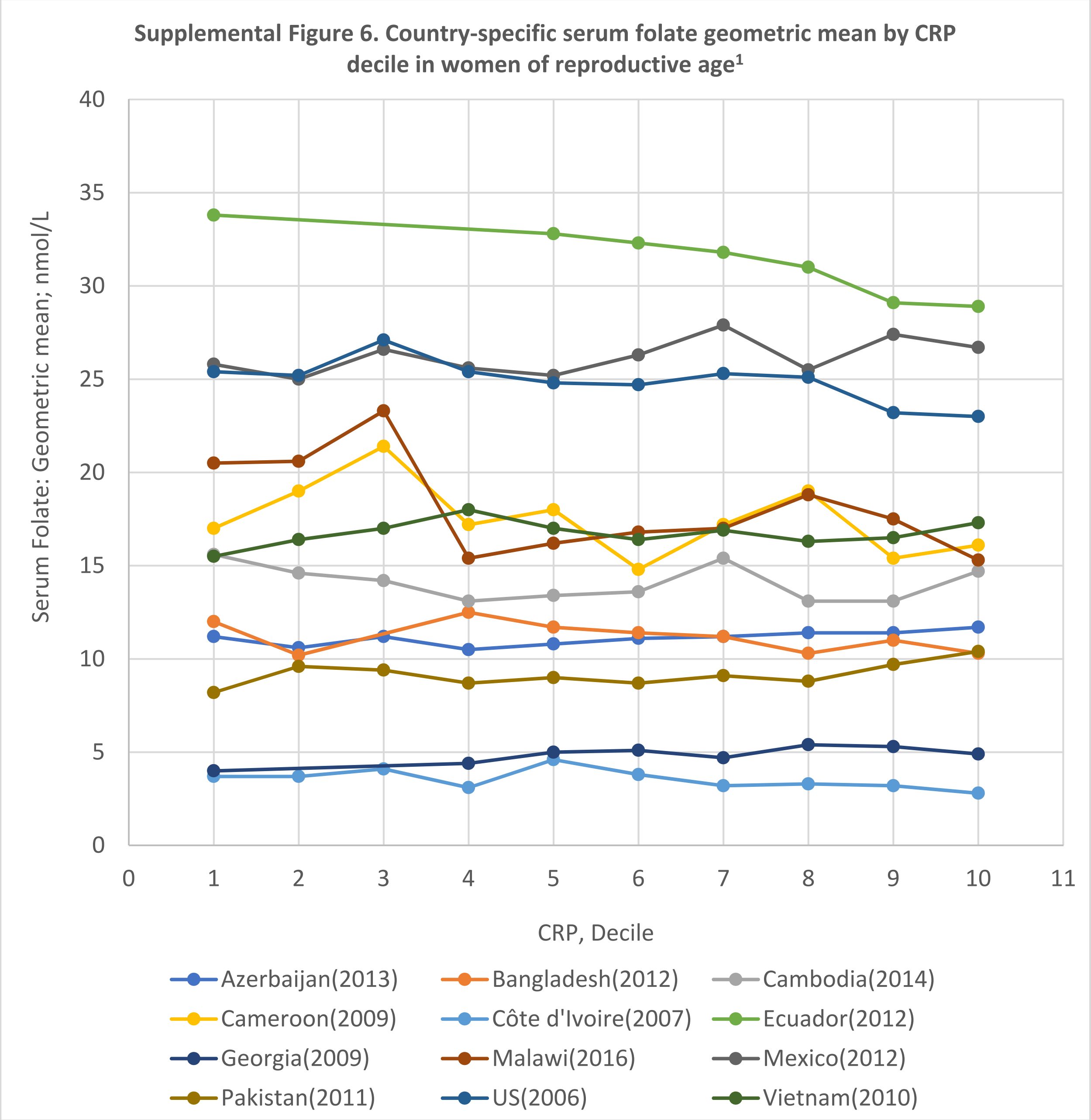

[1]alpha-1-acid glycoprotein: AGP; C-reactive protein: CRP

ONLINE SUPPORTING MATERIAL

Supplemental Figure 7. Country-specific serum folate geometric mean by AGP decile in women of reproductive age[1]

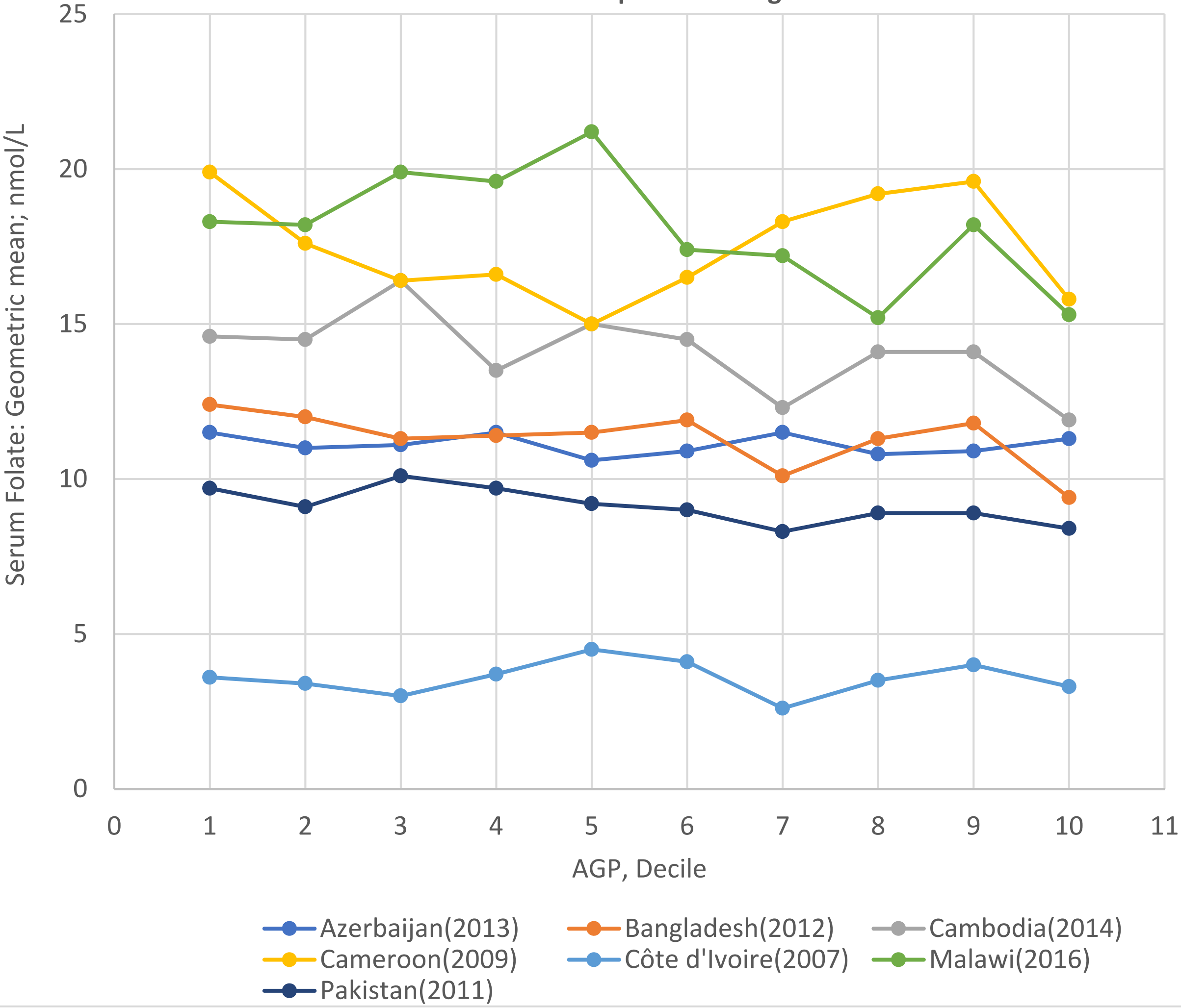

[1]alpha-1-acid glycoprotein: AGP; C-reactive protein: CRP

ONLINE SUPPORTING MATERIAL

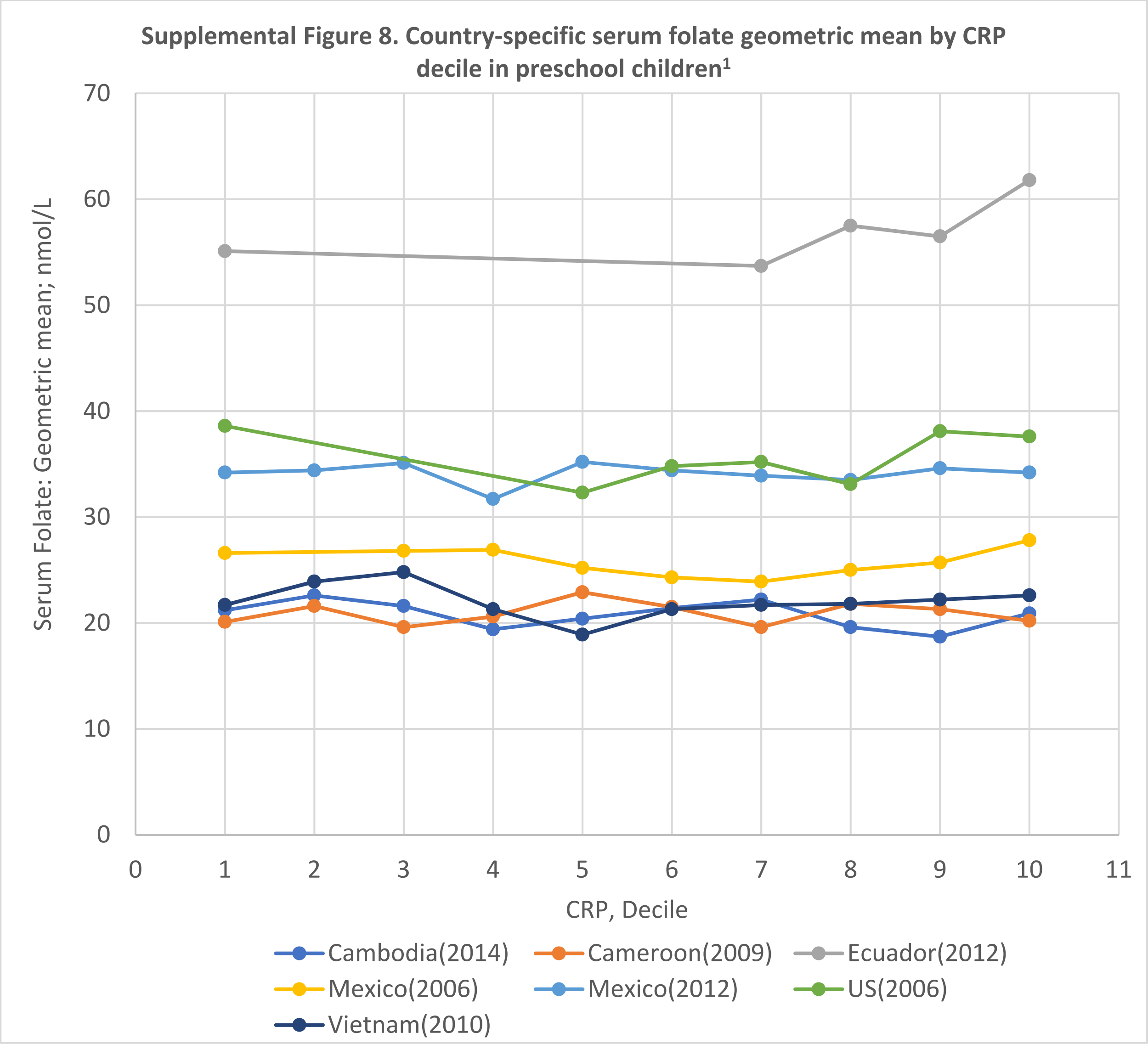

[1]alpha-1-acid glycoprotein: AGP; C-reactive protein: CRP

ONLINE SUPPORTING MATERIAL

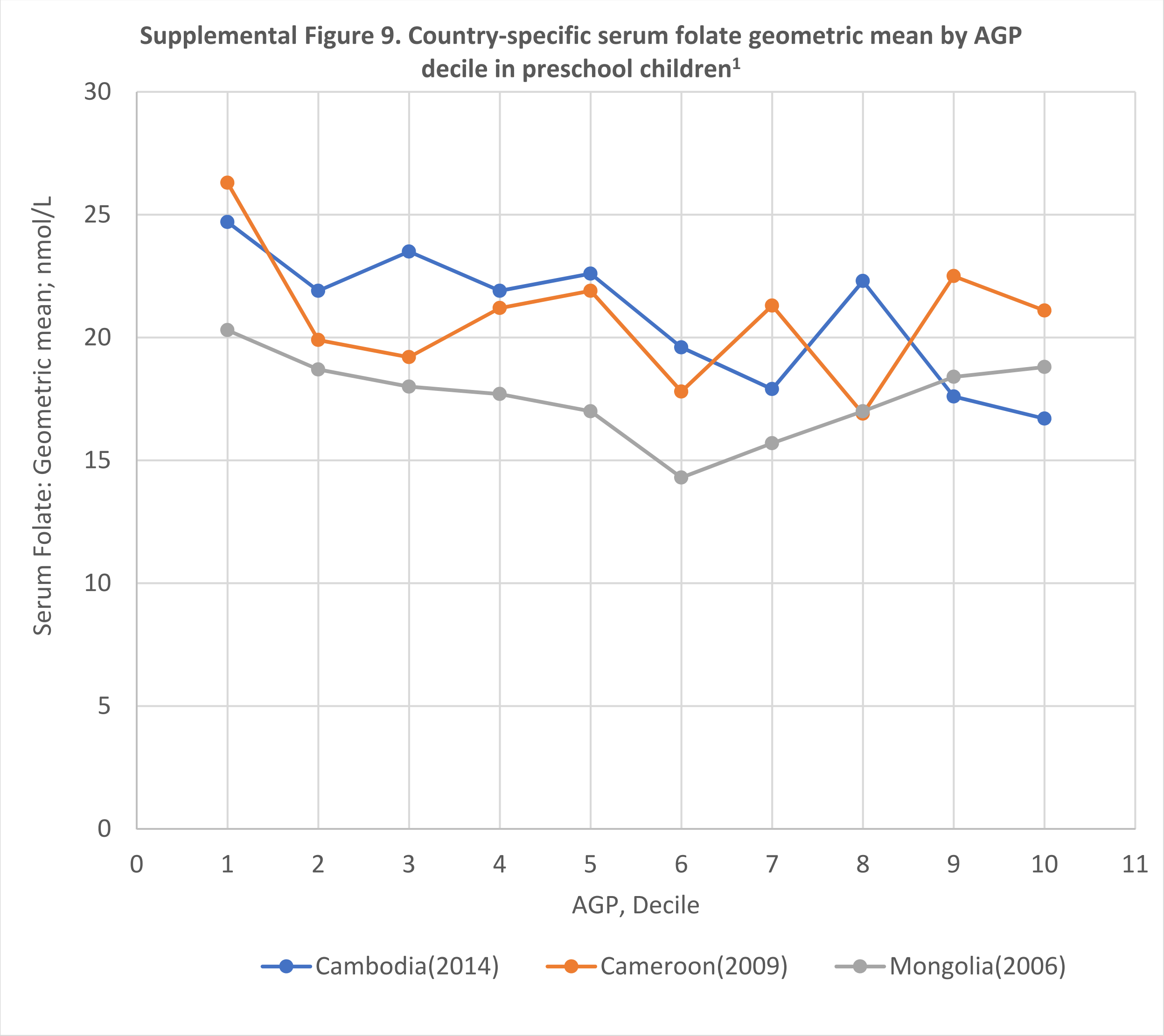

[1]alpha-1-acid glycoprotein: AGP; C-reactive protein: CRP

ONLINE SUPPORTING MATERIAL

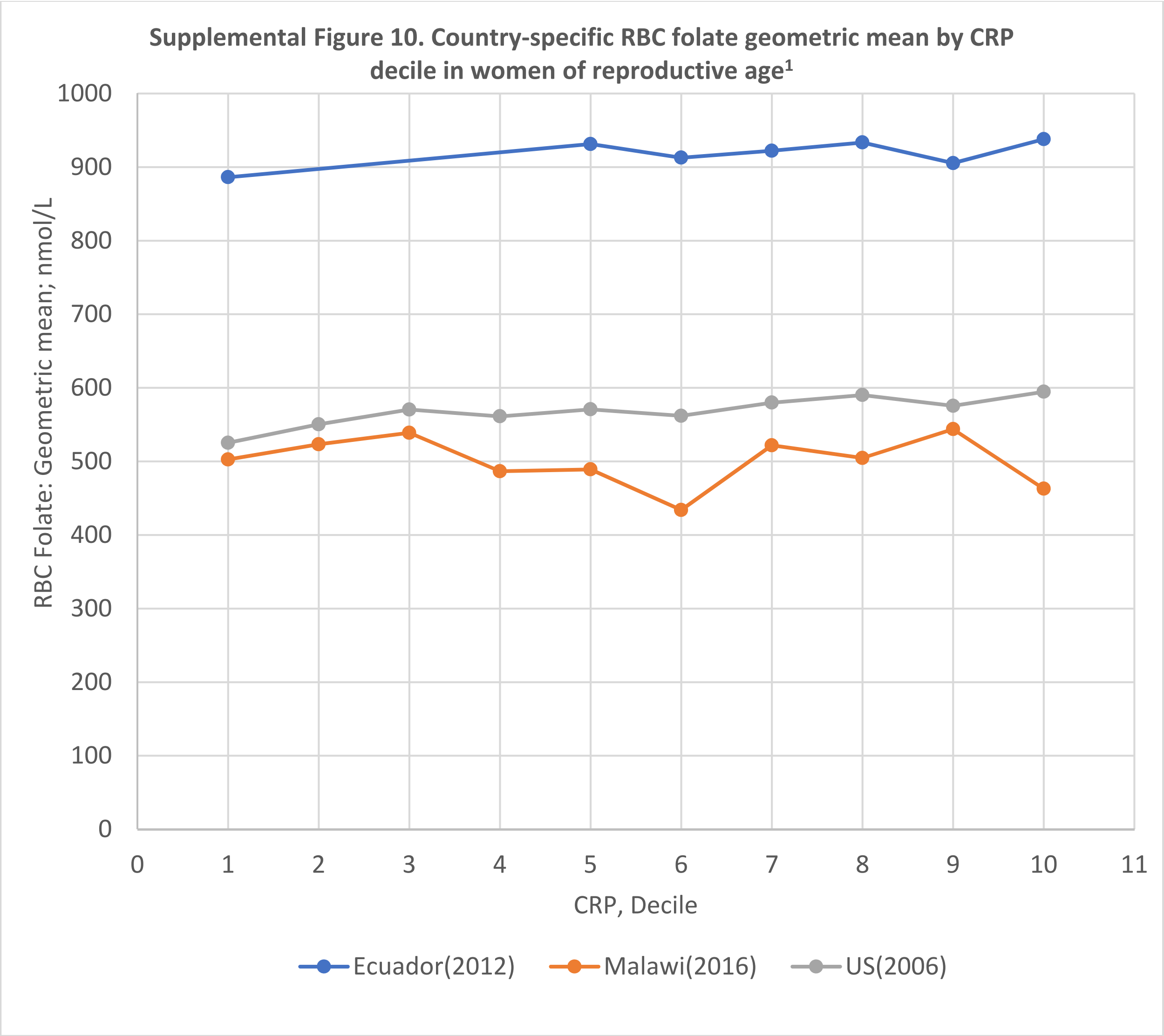

[1]alpha-1-acid glycoprotein: AGP; C-reactive protein: CRP

Supplemental Figure 11. Country-specific RBC folate geometric mean by AGP decile in women of reproductive age[1]

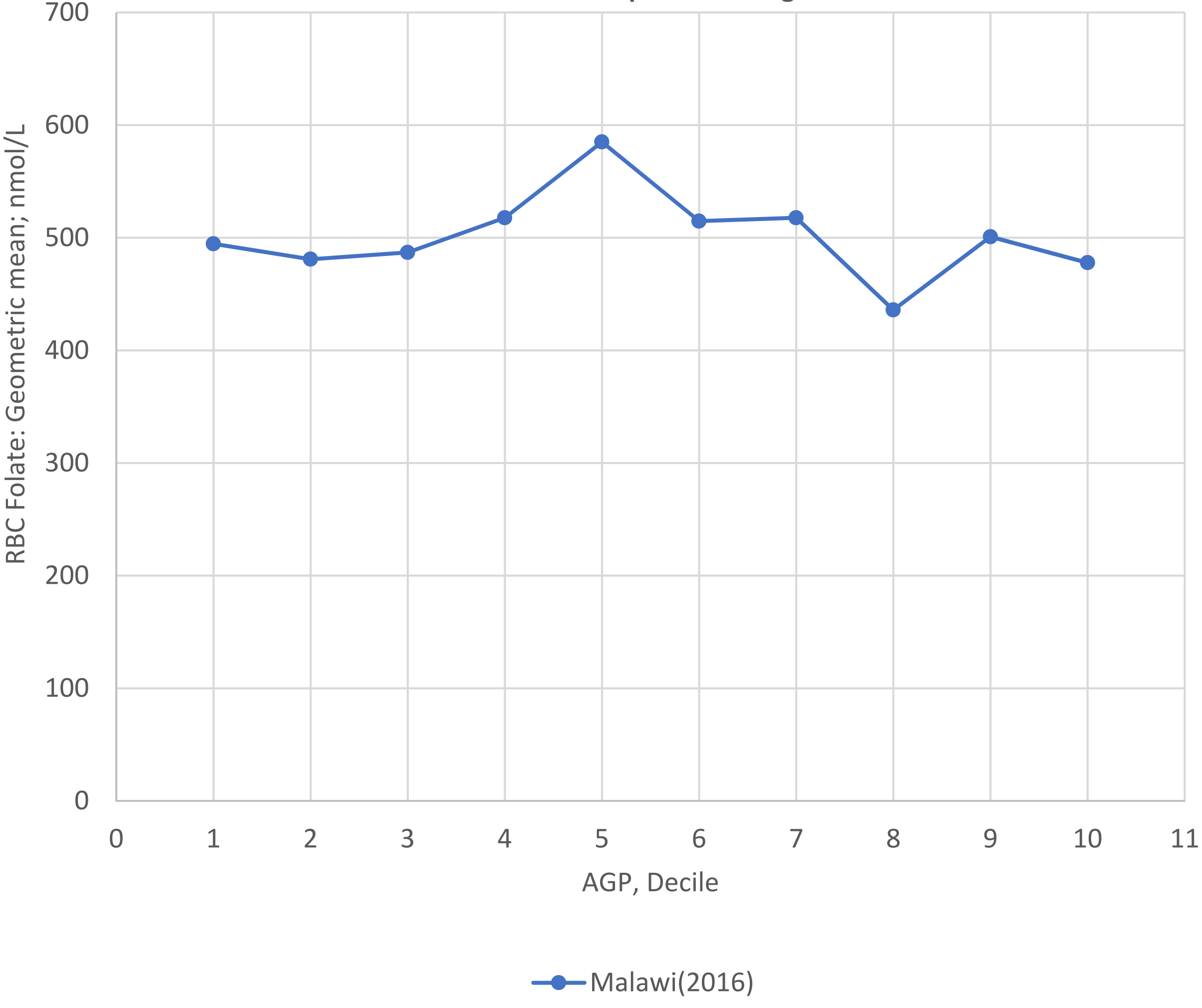

[1]alpha-1-acid glycoprotein: AGP; C-reactive protein: CRP

ONLINE SUPPORTING MATERIAL

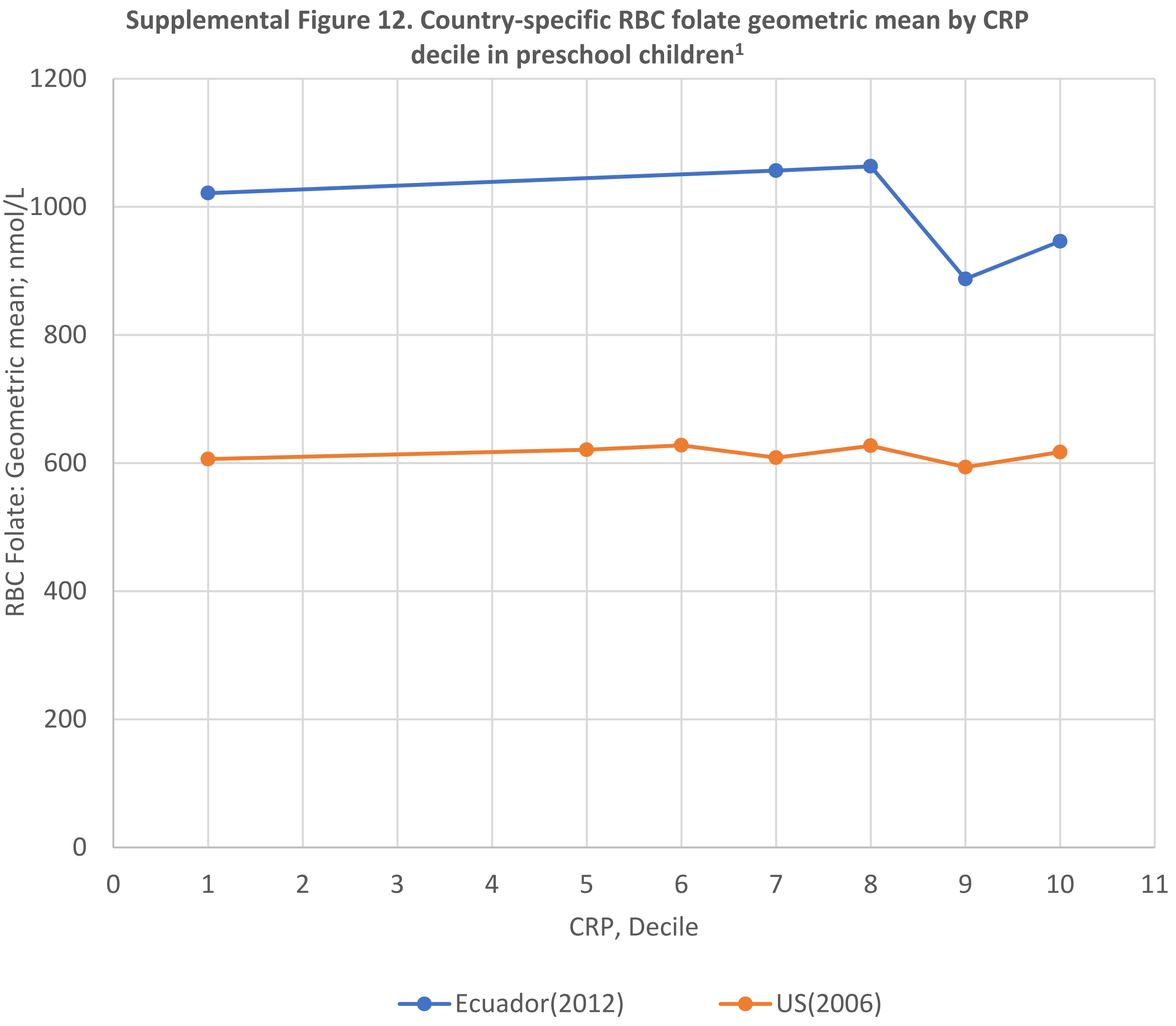

[1]alpha-1-acid glycoprotein: AGP; C-reactive protein: CRP

ONLINE SUPPORTING MATERIAL

Supplemental Table 1. Biomarker availability and lab methods by survey[1]

Survey, year	Vitamin B-12	Serum Folate	RBC Folate	CRP	AGP
Azerbaijan, 2013	Microbiologic assay	Microbiologic assay	-	ELISA	ELISA
Bangladesh, 2012	Immunoassay	Immunoassay	-	Sandwich ELISA	Sandwich ELISA
Cambodia, 2014	Immunoassay	Immunoassay	-	Sandwich ELISA	Sandwich ELISA
Cameroon, 2009	Radioassay	Radioassay	-	Sandwich ELISA	Sandwich ELISA
Colombia, 2010	Immunoassay	-	-	Turbidimetry	-
Côte d'Ivoire, 2007	Microbiologic assay	Microbiologic assay	-	Sandwich ELISA	Sandwich ELISA
Ecuador, 2012	Immunoassay	Immunoassay	Immunoassay	Nephelometry	-
Georgia, 2009	-	Microbiologic assay	-	Turbidimetry	-
Malawi, 2016	Immunoassay	Radioassay	Microbiologic assay	ELISA	ELISA
Mexico, 2006	Immunoassay	Immunoassay	-	Nephelometry	-
Mexico, 2012	Immunoassay	Immunoassay	-	Nephelometry	-
Mongolia, 2006	-	Microbiologic assay	-	--	Turbidimetry
Pakistan, 2011	Immunoassay	Immunoassay	-	Immunoassay	Turbidimetry
UK, 2014	Immunoassay	-	-	Immunoassay	-
United States, 2006	Radioassay	Radioassay	Radioassay	Turbidimetry	-
Vietnam, 2010	Microbiologic assay	Microbiologic assay	-	ELISA	-

[1] **Abbreviations:** Alpha-1-acid glycoprotein (AGP), C-reactive protein (CRP), red blood cell (RBC), Enzyme-linked immunosorbent assay (ELISA)

ONLINE SUPPORTING MATERIAL

Supplemental Table 2 Correlation between inflammation biomarkers and vitamin B-12, serum folate and RBC folate, stratified by malaria status by survey: the BRINDA project[1]

Survey	Malaria	Vitamin B-12					Serum folate					Red blood cell folate				
		n	CRP r	p[2]	AGP r	p	n	CRP r	p[2]	AGP r	p	n	CRP r	p[2]	AGP r	p
**Women of Reproductive Age**																
Côte d'Ivoire (2007)	Negative	368	0.16		0.14		736	-0.08		0.01		-	-	-	-	-
	Positive	23	0.17	0.94	0.18	0.63	39	-0.07	0.75	0.29	0.12	-	-	-	-	-
Cameroon (2009)	Negative	291	0.01		-0.05		291	-0.11		0.00		-	-	-	-	-
	Positive	42	0.07	0.63	0.08	0.34	42	0.02	0.73	-0.05	0.89	-	-	-	-	-
Malawi (2016)	Negative	633	0.04		0.05		624	-0.15		-0.12		626	-0.08		-0.06	
	Positive	111	-0.09	0.09	-0.21	0.02	111	-0.14	0.83	-0.14	0.76	110	-0.06	0.61	-0.10	0.81
**Preschool Children**																
Cameroon (2009)	Negative	263	0.03		-0.04		263	0.06		0.00		-	-	-	-	-
	Positive	91	-0.12	0.72	-0.11	0.46	92	0.03	0.80	-0.07	0.43	-	-	-	-	-

[1] **Abbreviations:** Biomarkers Reflecting Inflammation and Nutritional Determinants of Anemia (BRINDA),Alpha-1-acid glycoprotein (AGP), C-reactive protein (CRP), preschool children (PSC), women of reproductive age (WRA), red blood cell (RBC)

[2] p value was calculated from T test in regression that took into account complex sampling effects (cluster, strata and sampling weight).Inclusion criteria were non-missing nutritional biomarker measures, malaria status and CRP and/or AGP measures. The sample size for CRP and AGP anlayses were the same by survey and nutritional biomarker, so only one n column was listed under each nutritional biomarker. P-value for interaction is reported, and interaction effect was tested in a regression model that regressed ln-transformed inflammation biomarker, malaria status and ln-transformed inflammation biomarker * malaria status on ln-transformed nutritional biomarker. Complex sampling design effects (cluster, strata and sampling weights) were taken into account.

ONLINE SUPPORTING MATERIAL

Supplemental Table 3: Weighted Spearman correlations between vitamin B-12 and CRP in preschool children and non-pregnant women of reproductive age, BRINDA project[1,2,3]

Survey	n	Vitamin B-12 * CRP		Vitamin B-12 * CRP[3]	
		r	P	r	p
**PSC**					
Cambodia	645	-0.01	0.75	-0.07	0.17
Cameroon	361	0.00	0.97	0.006	0.91
**WRA**					
Azerbaijan	1317	-0.02	0.63	-0.02	0.53
Cambodia	700	-0.01	0.84	0.03	0.69
Cameroon	335	0.01	0.80	0.02	0.65
Côte d'Ivoire	400	0.16	0.01	0.15	0.01
Malawi	765	0.01	0.74	-0.006	0.89

[1] alpha-1-acid glycoprotein (AGP), Biomarkers Reflecting Inflammation and Nutritional Determinants of Anemia (BRINDA), C-reactive protein (CRP)

[2] p value was calculated from T test in regression model that took into account complex sampling (cluster, strata and sampling weight).

[3]CRP values below the LOD (0.5) were assigned a value of 0.25. All laboratory analyses were conducted using sandwich ELISA of VitMin lab

ONLINE SUPPORTING MATERIAL

Supplemental Table 4: Weighted Spearman correlations between serum folate and CRP in preschool children and non-pregnant women of reproductive age, BRINDA project[1,2,3]

Country		SFO*CRP		SFO*CRP[3]	
	n	r	p	r	p
**PSC**					
Cambodia	646	-0.05	0.32	-0.02	0.67
Cameroon	362	0.00	0.95	0.007	0.92
**WRA**					
Azerbaijan	2551	0.07	0.01	0.07	0.01
Cambodia	699	-0.07	0.20	-0.05	0.31
Cameroon	335	-0.06	0.33	-0.08	0.22
Côte d'Ivoire	792	-0.09	0.003	-0.095	0.002
Malawi	755	-0.13	0.01	-0.12	0.02

[1] alpha-1-acid glycoprotein (AGP), Biomarkers Reflecting Inflammation and Nutritional Determinants of Anemia (BRINDA), C-reactive protein (CRP)

[2] p value was calculated from T test in regression model that took into account complex sampling (cluster, strata and sampling weight). Mongolia did not apply complex survey design.

[3]CRP values below the LOD (0.5) were assigned a value of 0.25. All laboratory analyses were conducted using sandwich ELISA of VitMin lab

ONLINE SUPPORTING MATERIAL

Supplemental Table 5: Weighted Spearman correlations between RBC folate and CRP in non-pregnant women of reproductive age, BRINDA project[1,2,3]

Country		RBC*CRP		RBC*CRP[3]	
	n	r	p	r	p
**WRA**					
Malawi	752	-0.06	0.28	-0.04	0.37

[1] alpha-1-acid glycoprotein (AGP), Biomarkers Reflecting Inflammation and Nutritional Determinants of Anemia (BRINDA), C-reactive protein (CRP)

[2] p value was calculated from T test in regression model that took into account complex sampling (cluster, strata and sampling weight). Mongolia did not apply complex survey design.

[3]CRP values below the LOD (0.5) were assigned a value of 0.25. All laboratory analyses were conducted using sandwich ELISA of VitMin lab
